# DICLOFENAC: NEW DATA ON CHRONIC TOXICITY AND BIOCONCENTRATION IN FISH

**DOI:** 10.1002/etc.2085

**Published:** 2013-01-16

**Authors:** Ulrich Memmert, Armin Peither, Roland Burri, Klaus Weber, Thomas Schmidt, John P Sumpter, Andreas Hartmann

**Affiliations:** † Eurofins RegulatoryRheinfelden, Switzerland; ‡ Harlan LaboratoriesItingen, Switzerland; § Institute for the Environment, Brunel UniversityUxbridge, United Kingdom; ‖ Novartis Pharma SteinStein, Switzerland

**Keywords:** Diclofenac, NSAID, Fish toxicity, Fish early life stage, Bioconcentration

## Abstract

Diclofenac (DCF) is a widely used nonsteroidal anti-inflammatory drug that is regularly detected in surface waters. To support a robust aquatic risk assessment, two early life stage (ELS) tests, compliant with the Organisation for Economic Co-operation and Development (OECD) test guideline 210, were conducted in rainbow trout and in zebrafish. Population relevant endpoints, such as hatching, growth, and survival, and in the trout study, histopathological effects in potential target organs, were examined. The bioconcentration of DCF in rainbow trout was measured in a separate study according to OECD test guideline 305. The bioconcentration factor (BCF) in rainbow trout remained below 10, demonstrating no relevant bioconcentration of DCF in fish. In the rainbow trout ELS test, the no observed effect concentration (NOEC) including histopathology was 320 µg/L. The effect of DCF on zebrafish growth was less clear, and the NOEC can be interpreted as 10 µg/L. However, for a number of reasons, the authors consider the moderately reduced growth of zebrafish exposed to concentrations of up to 320 µg/L not a repeatable, treatment-related effect of DCF. This leads us to a conclusion that DCF has, with high probability, no adverse effect on both fish species up to 320 µg/L. This NOEC indicates a sufficient safety margin for fish populations, because concentrations of DCF in European rivers are in the range of ng/L to low µg/L. Environ. Toxicol. Chem. 2013;32:442–452. © 2013 SETAC

## INTRODUCTION

Diclofenac (DCF) is an important nonsteroidal anti-inflammatory drug (NSAID) and is used for the treatment of painful and inflammatory conditions. It has a well-known safety and efficacy profile. Diclofenac is the active ingredient in many oral as well as topical formulations (such as creams) available to patients under both prescriptions as well as over-the-counter products. The estimated overall yearly worldwide consumption of DCF as a human and veterinary pharmaceutical drug is >1000 tons/year 1.

Diclofenac can be detected in effluents of wastewater treatment plants (low µg/L range) and the aquatic environment (ng/L to low µg/L range) in many countries 1–3. Although the removal efficiency varies, sewage treatment plants are generally not able to remove DCF to a high extent, as both the biodegradability and the elimination by adsorption of DCF to activated sludge is low (reviewed by Zhang et al. 1 and Pal et al. 4). Acute testing resulted in relatively low toxicity of DCF for fish, daphnia, and algae, with no observed effect concentrations (NOEC's) or effective concentration for 10% (EC10) >1.0 mg/L and effective concentration for 50% (EC50) >10 mg/L 5–9. However, after chronic exposure, several authors observed histopathological effects in trout species at relatively low concentrations around 1.0 to 5.0 µg/L 10–13. These findings induced a public concern regarding a risk of DCF to fish populations.

The seemingly high octanol-water partition coefficient *K*_OW_ of DCF (log *K*_OW_ 4.51; 14) also caused concerns regarding a high bioconcentration in fish and a subsequent potential for secondary poisoning within the aquatic food chain and to fish-eating birds. A significant bioconcentration of DCF was measured in some fish organs 10. These findings of chronic toxicity and bioconcentration in fish are currently the main basis for many stakeholders to conclude that even the low concentrations of DCF measured in surface waters present a high risk to the environment. Hence, there is a need for high quality ecotoxicological data from long-term exposure studies with DCF to allow for a robust environmental risk assessment. For this purpose, the warm water fish species zebrafish (*Danio rerio*) and the cold water species rainbow trout (*Oncorhynchus mykiss*) were tested in fish early life stage tests following Organisation for Economic Co-operation and Development (OECD) test guideline 210 15. Histopathology of the potentially targeted organs (kidney, liver, and gills) was additionally conducted in *O. mykiss* to verify earlier pathological findings and to allow the translation of adverse histological effects to population-relevant study endpoints like development, growth, and survival of fish within one study. Additionally, the bioconcentration of DCF was studied in *O. mykiss,* by using the internationally accepted OECD test guideline 305 16.

## MATERIALS AND METHODS

### Test organisms and acclimation

Fertilized eggs for the early life stage (ELS) test with rainbow trout were obtained from a professional trout breeder (Störk, Bad Saulgau, Germany). Zebrafish eggs were collected from our own long-standing breeding culture (originally obtained from West Aquarium, Bad Lauterberg, Germany). All eggs were acclimated to the test water before the start of the exposure. The time period between egg fertilization/collection and test start was very short: approximately 4 h for trout and 3 h for zebrafish, respectively.

For the bioconcentration study, juvenile rainbow trout were used, which were raised in our facility from eggs that were obtained from a professional breeder (Hohler, Zeiningen, Switzerland). The fish were acclimated to the test water and test conditions for at least two weeks before the test start. The average weight of the test fish, calculated per test vessel, ranged from 1.1 to 1.2 g at the start of the bioconcentration study, as required by OECD test guideline 305 16.

### Test water

Both tests with rainbow trout (ELS and BCF study) were run in local tap water (nonchlorinated well water of drinking water quality). Reconstituted test water was used for the zebrafish study, consisting of analytical grade salts dissolved in purified water based on ISO standard 6341 17. Water temperature, pH, and dissolved oxygen were measured at the start and end of all tests, and at least once per week during the study periods, by calibrated electrodes. Water hardness and total organic carbon (TOC) concentration in the water are listed in Table 1.

**Table 1:** Study parameters

	Fish early life stage tests				Bioconcentration test	
		
	Rainbow trout		Zebrafish		Rainbow trout	
			
	Prehatch period	Posthatch period	Prehatch period	Posthatch period	Exposure period	Depuration period
Experimental days	0–33	34–95	0–4	5–34	0–14	15–28
Water temperature (°C)	10.3–10.7	13.2–14.4	26.5–26.6	26.0–26.7	13.5–15.0	13.3–15.1
pH	7.7–7.8	7.6–7.9	7.0–7.2	7.0–7.2	7.5–8.4	7.4–8.0
Dissolved oxygen concentration (mg/L)	9.4–9.9	7.5–9.6	8.0–8.1	7.3–9.8	8.4–10.6	8.4–9.3
Total hardness (mg/L)	125		193		180	
Total organic carbon (TOC) concentration (mg/L)	Not measured		Not measured		0.00–0.70	0.01–0.35
Loading rate (g fish/L/d)	0.18		0.06		0.38	

### Test substance

Diclofenac sodium salt (CAS-ID: 15307-79-6; purity: 100.1%; provided by Novartis) was used for all tests. As recommended by OECD test guideline 305, radiolabeled test material ([^14^C]DCF sodium salt, ring labeled, specific radioactivity: 3.603 MBq/mg (salt); radiochemical purity: 98.5%; provided by Novartis) was added in the BCF study for analytical purposes.

### Study design

All studies were conducted according to Good Laboratory Practice (GLP) in a laboratory certified by AAALAC International (Association for Assessment and Accreditation of Laboratory Animal Care). Temperature-controlled conditions (Table 1) and a 16 h light to 8 h dark photoperiod were applied. The nominal test concentrations in the ELS test with rainbow trout were 3.2, 10, 32, 100, 320, and 1000 µg/L. In the zebrafish ELS study, a slightly higher concentration range of nominal 10, 32, 100, 320, 1000, and 3200 µg/L was tested, because a range-finder pretest showed no mortality in zebrafish embryos at 1500 µg/L after 5 d of exposure. Both ELS studies included a nontreated control. Test duration of the trout ELS test was in total 95 d, with a prehatch period from day 0 to 33 followed by a 62-d posthatch period. The test period of the zebrafish ELS study was 34 d in total, with a 4-d prehatch period and 30 d posthatch.

All tests were run under flow-through conditions. Diclofenac sodium salt was dissolved in aqueous application solutions without any auxiliary solvent. The application solutions were light-protected and continuously dosed by a timer-controlled digital dispenser (Hamilton) into mixing vessels with continuous stirring and constant test water inlet. The resulting test solutions flowed directly into the test vessels (BCF study) or were divided for the ELS tests into four replicate test tanks per test concentration (in the trout ELS study by electronically regulated splitting devices [Pequitec], in the zebrafish ELS test by peristaltic pumps). The flow of the test solutions assured an at least sixfold water renewal per day in the test vessels of the ELS tests, and an at least 3.3-fold renewal per day in the BCF test. Application solutions in the ELS tests were renewed every 7 to 8 d.

Both ELS studies were run with four tank replicates (glass aquaria) per test concentration and control, and included 15 eggs per replicate. The rainbow trout eggs were placed in stainless steel egg cups with a stainless steel net bottom. The egg cups were slowly moved up and down using a motorized rocker arm to assure a nonturbulent circulation of the test media through the mesh bottom. After hatching, the trout larvae were released into the 13 L test vessels. In the ELS test with zebrafish, the eggs were placed directly into the test vessels. The size of the glass vessels (200 ml up to 5 L) was adapted as the fish grew. The four test vessel replicates were positioned in a flow-through aquarium. The test solutions circulated through the four test vessels, whereby the larvae and juvenile fish were kept back in the test vessels by a stainless steel net. The larvae and juvenile fish in the ELS tests were fed ad libitum, the trout with commercial dry food (Hokovit, 10.3% lipid + 49.9% total protein; Hoffmann), the larvae and juvenile zebrafish with living ciliates (*Paramecium* spec., own culture), commercial fish dry food (Tetra), and/or *Artemia salina* (own culture).

In the BCF flow-through test, application solutions with mixed amounts of [^14^C]DCF and nonradiolabeled DCF, dissolved in deionized water, were used for the dosage to achieve nominal concentrations of 2.0 µg/L at 0.80 MBq/mg and 20 µg/L at 0.41 MBq/mg, respectively.

During the accumulation period, one tank served as the control and two tanks received the test substance from the application solutions delivered via a dispenser unit (Hamilton) as described above. Each of the test tanks (glass aquaria) contained 75 L of water, with 70 fish (low and high dose, each) and 45 fish (control). The size of the control group was kept as low as possible to meet animal welfare requirements. The test fish were fed daily with commercial fish food Hokovit (see above) and at a ratio corresponding to approximately 2 to 3% of the average fish body weight, taking into account increasing body weights and the decreasing number of fish per sampling interval.

### Analytical determination of DCF

In both ELS tests, analytical samples were typically taken once or twice a week from all test solutions (for dates of analyses refer to Supplemental Data, Tables S1 and S2). The stability of DCF in the application solutions was analytically confirmed. All analytical samples were diluted 1.4-fold with methanol before storing them deep-frozen and light protected. Immediately before analyses the samples were thawed at room temperature and shaken manually to obtain homogeneous solutions. Low-level test concentrations (3.2 µg/L to 32 µg/L) were determined by high performance liquid chromatography-mass spectrometry (HPLC-MS/MS) using external calibration. The limit of quantification (LOQ) of this method was 1.4 µg DCF/L in the zebrafish and 2 µg DCF/L in the trout study. High-level test concentrations (100 µg/L to 1000 µg/L) and the application solutions were analyzed by HPLC with ultraviolet (UV) detection using external calibration. Injected samples were quantified by peak areas with reference to the respective calibration curve. The absence of impurities was confirmed by the chromatographic profile. For detailed information on the analytical methods and their validations, see Supplemental Data.

In the BCF study, water samples were taken from the mixing chambers and from the test solutions every day, and the total radioactivity was measured by liquid scintillation counting (LSC) (see Supplemental Data for details). The DCF dosage was adjusted if necessary. Apart from total radioactivity, the parent substance [^14^C]DCF and all other radioactive fractions were analyzed in the test solutions at five dates during the exposure period using HPLC (see Supplemental Data for details). As six C-atoms in the ring structure of DCF were labeled and the ring structure is conserved in all primary DCF metabolites, as described by Kallio et al. 18 and Lahti et al. 19, total radioactivity can be expected to include parent DCF and all DCF metabolites. Fish were sampled on five dates during the accumulation phase and on four dates during the depuration phase. Four fish were randomly selected per sampling date, sacrificed in 1.5% (v/v) 2-phenoxy-ethanol, and blotted dry. The fish were weighed and completely solubilized with tissue solubilizer (PerkinElmer, ∼100 mg/ml solubilizer). Thereafter, duplicate solubilized subsamples (corresponding to 100 or 200 mg per fish) were measured by LSC (for details see Supplemental Data).

### Evaluation and statistics

In the ELS tests, the hatching of larvae was recorded each working day. Percentage of hatching success was calculated for each tank replicate by dividing the number of hatched larvae by the number of inserted eggs. The development rate was calculated as the reciprocal of the hatching time (unit: 1/d), representing the embryo development per day until the day of hatch. Differences in hatching rate were evaluated by Fisher's exact binomial test, those of the development rate by the Dunnett *t* test (trout) or Welch test (zebrafish), both tests one-sided smaller with *p* ≤ 0.05. The survival rate of the test fish was calculated for each tank replicate by dividing the number of surviving fish until test end by the number of larvae hatched. Differences in fish mortality between control and treatments were evaluated by Fisher's exact binomial test (one-sided greater, *p* ≤ 0.05). Fish total body length (mouth to end of tail fin) and body wet weight were determined for every individual fish. Body dry weight measurements (total dry weight of all fish per tank replicate) were only possible for zebrafish because the trout bodies were needed for the histopathological evaluation. Length and weight were determined on the tank replicate basis, and median values per replicate were used to avoid any bias caused by single extremely small or large individuals. The mean values given in the present publication refer to the arithmetic mean and standard deviation (SD) of the median values of the four replicates per treatment. Differences in mean length and body weight were evaluated by Dunnett *t* tests (one-sided smaller, *p* ≤ 0.05).

After determination of the fish wet weight at test termination, all rainbow trout were sectioned to allow the fixative to penetrate the body of the fish and fixed in Davidson's fixative for histopathology. A total of 20 fish (five per tank replicate) from each test concentration and from the control were randomly selected for histopathological examinations. These fish were trimmed in a sagittal direction; liver, kidney, and gills were present in these samples. The samples were processed, embedded in paraffin wax, and cut in longitudinal direction at a nominal thickness of 2 to 4 µm. One section was stained with haematoxylin and eosin, another section by Azan Heidenhains stain. The sections were evaluated by light microscopy. For details on the identified symptoms refer to Supplemental Data. All symptoms were archived as digital pictures. The symptoms were categorized by incidence, that is, number of fish affected and by severity (grade 1 = < 10% [minimal], grade 2 = 10 to 39% [slight], grade 3 = 40 to 59% [moderate], grade 4 = 60 to 79% [marked], grade 5 = 80 to 100% [severe], based on estimated percentage related to tissue area present for evaluation). Lesions consisting of necrosis and inflammations were considered to be the most important ones affecting the test organisms. The possible impact of a finding on the test fish was determined by expert judgment by an experienced specialist. Statistical evaluation of all lesions was carried out according to the Armitage trend test 20, by using all treatment groups (control and the six test concentrations) and all groups without the highest treatment group, and by the Fisher's exact test.

In the bioconcentration study, the following three BCFs were calculated according to OECD guideline 305: (1) steady state BCF_SS_ based on the concentration in fish at the plateau level, which means, at the last three successive fish sampling dates; (2) kinetic BCF_K_ obtained by the ratio of the uptake rate constant (k_1_) to the depuration rate constant (k_2_); and (3) BCF_L_ (BCF_SS_ normalized to 5% fish lipid content according to the OECD 21, see Supplemental Data for details). All BCF's were based on total radioactivity in parent equivalents (µg equivalents/g) in fish and the average measured concentration of total radioactivity in water. For BCF_L_ the mean lipid content in control fish was determined: 20 fish were sampled (10 individuals on day 4, and 10 on day 14 of the uptake phase). The fish of each sampling were pooled and the lipid content was measured by the accelerated solvent extraction method (for details see Supplemental Data).

## RESULTS

### Validity criteria of the OECD test guidelines

All validity criteria mentioned in the test guidelines OECD 210 and 305 were fulfilled. For the ELS tests, criteria were as follows: dissolved oxygen concentration in test media ≥60% of saturation; difference in water temperature ≤ ±1.5°C between aquaria or between successive days; hatching success in the control ≥66%; and posthatch success in the control ≥70%. For the BCF test, criteria were as follows: temperature variation ≤ ±2°C; concentration of dissolved oxygen ≥60% of saturation; concentration of the test substance within ±20% of the mean measured values during the uptake phase; and mortality or other adverse effects in both control and treated fish <10% at the end of the test.

### ELS test with rainbow trout

#### Analytics

To confirm the correct dosage of DCF during the test, 77 test solution samples taken at up to 22 dates throughout the test were analyzed (for details see Supplemental Data, Table S1). Diclofenac was not detectable in the control samples. The DCF concentrations in the analyzed test media samples of the concentrations from 3.2 to 1000 µg/L were in the range of 78 to 147% of the nominal values during the test period. Because not all analytical measurements were in the range of 80 to 120% of nominal (required by OECD test guidelines to relate the effects to nominal concentrations), the biological results were based on the nominal concentrations and the NOEC's additionally on the mean measured DCF concentrations (for mean measured values see Supplemental Data, Table S1). The two lowest test concentrations of 3.2 and 10 µg/L were analyzed only on days 0 and 4, because these low concentrations were far below the NOEC and thus not relevant for the interpretation of the biological results.

#### Water parameters

Temperature, pH, dissolved oxygen, and so on, measured during the studies are listed in Table 1.

#### Hatching rate

**Fig. 1:**
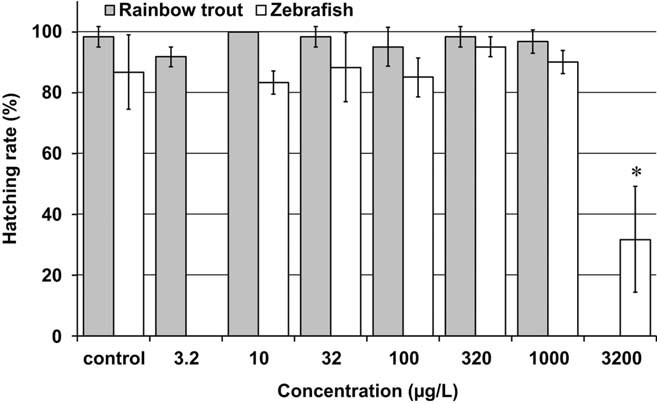
Effect of diclofenac on hatching rate in the early life stage tests. Means ± standard deviations are plotted. Asterisk (*) equals statistically significant to the control (Fisher's exact binomial test on egg mortality, one-sided greater, *p* ≤ 0.05).

The hatching of the trout larvae from the eggs was completed on day 33 postfertilization. The mean hatching rate in the control was 98 ± 3% (mean ± SD, Fig. 1). For detailed results of all population parameters, see also Supplemental Data, Tables S3 and S4. At all test concentrations, the mean hatching rates were nearly equal to or even slightly higher compared to the control (93 to 102% of control value) and statistically did not differ significantly from the control. The NOEC for the hatching rate was therefore determined at ≥1084 µg/L (nominal ≥ 1000 µg/L).

#### Development rate

First larvae were observed at day 29 postfertilization in the control and the test concentrations of nominal 10 and 320 µg/L. The major part of the larvae hatched at day 32 at all treatments, including the control. The development rate of the larvae during the hatching period was comparable at all test concentrations, however, statistically it was significantly lower at the lowest test concentration compared to the control (97% of control). Because no clear dose-response was observed at the large test concentration range above 3.2 µg/L, the reduced development rate in the lowest test concentration was found to be caused by natural variability, and not attributed to DCF treatment. Therefore, the NOEC for egg development rate was determined at ≥1084 µg/L.

#### Survival

**Fig. 2:**
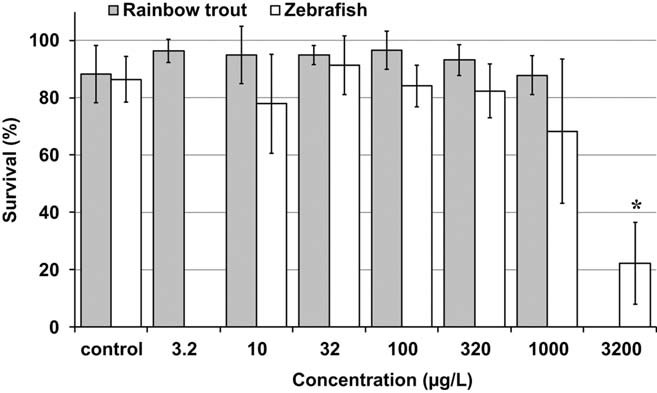
Effect of diclofenac on survival from hatch until the end of the early life stage tests. Means ± standard deviations are plotted. Asterisk (*) equals statistically significant to the control (Fisher's exact binomial test on fish mortality, one-sided greater, *p* ≤ 0.05).

The survival rate of the control fish until the end of the test was 88 ± 10% (mean ± SD), demonstrating suitability of the test conditions. The mean survival rates at all test concentrations were in the range of 100 to 109% of the control value and statistically did not significantly differ from the control (Fig. 2). All fish were healthy and showed normal behavior and no visible abnormalities. The NOEC for survival was therefore determined at ≥1084 µg/L.

#### Growth

**Fig. 3:**
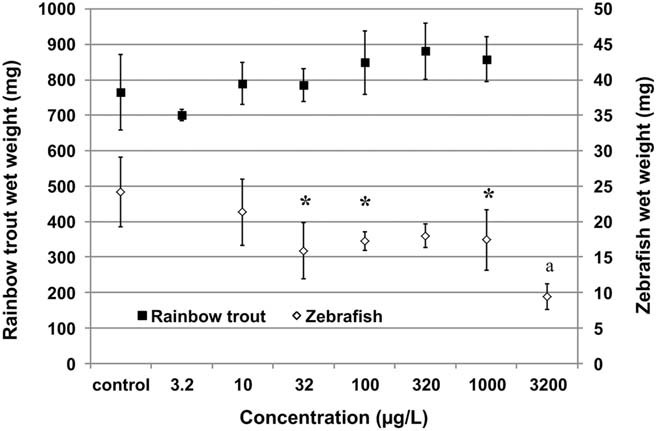
Effect of diclofenac on fish wet weight at the end of the early life stage tests. Medians of body wet weight were calculated per tank replicate to reduce the impact of individual outliers. The arithmetic mean ± standard deviation as shown in this figure was calculated from the median values of the four replicates per treatment. Asterisk (*) equals statistically significant to the control (Dunnett *t* test, one-sided smaller, *p* ≤ 0.05); a = treatment excluded from statistical analysis due to low number of 1 to 3 fish per tank replicate (fish growth in tank replicates with significant mortality should not be included in the data evaluation according to OECD test guideline 215 33).

The mean body length of the trout in all test concentrations ranged from 96 to 105% of the control value and statistically did not differ significantly from the control. The trout wet weights at all test concentrations were in the range of 92 to 115% of the control value and also statistically not significantly different to the control (Fig. 3). The NOEC for the growth of rainbow trout in terms of body length and weight was therefore ≥1084 µg/L (nominal ≥1000 µg/L).

#### Histopathology

**Fig. 4:**
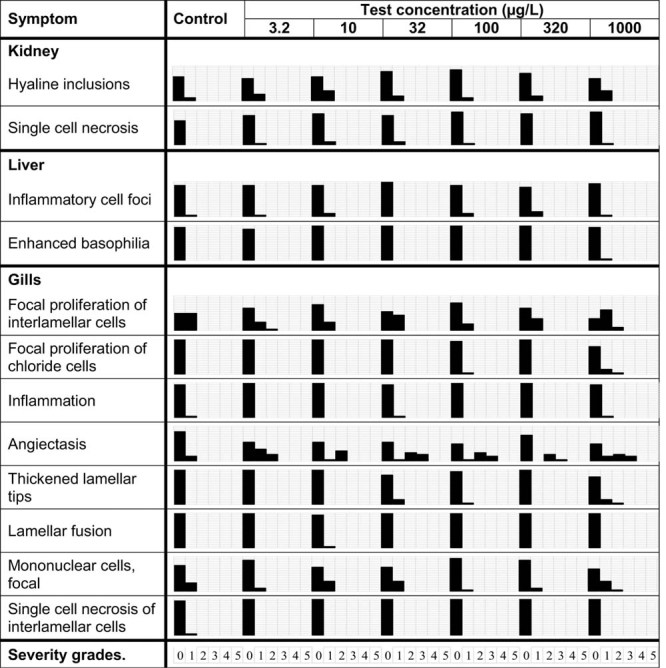
Incidence and severity of histopathological symptoms in kidney, liver, and gills of rainbow trout at the end of the early life stage test with diclofenac. Incidence is indicated by vertical bar height, severity grade (0 to 5) is displayed horizontally. Severity grade 0 = no incidence; grade 1 = < 10% (minimal); grade 2 = 10 to 39% (slight); grade 3 = 40 to 59% (moderate); grade 4 = 60 to 79% (marked); grade 5 = 80 to 100% (severe).

Hyaline inclusions, single cell necrosis in kidney, inflammatory cell foci, and enhanced basophilia in liver, respectively, were only at severity grade 1 (minimal) at all concentrations up to the highest test concentration. Minimal severity grade observations were also seen in some of the control fish. The incidence of these symptoms was randomly distributed throughout the treatment groups without a concentration-effect relationship (Fig. 4). The findings in kidney and liver were minimal and within the range of normal background alterations. Therefore, both organs were found to not be adversely affected by the DCF treatments. The overall symptoms observed in the gills were considered to show slight adverse effects only at the nominal concentration of 1000 µg/L. Minimal to slight degenerative lesions were recorded at this highest treatment. These lesions consisted of increasing incidences and/or severities of focal proliferation of interlamellar and chlorine cells, associated with thickened lamellar tips. Also, mononuclear cell foci increased slightly in severity at 1000 µg/L. However, all findings were recorded only at minor severity degrees (at maximum grade 2). The Fisher's exact test showed no significant increase in incidence (*p* > 0.05) for these lesions up to and including 1000 µg/L. However, an effect at the highest test concentration was revealed by the Armitage trend test: a statistically significant (*p* < 0.05) trend for increase in incidence and severity grade was induced for focal proliferation of interlamellar and chlorine cells and thickened lamellar tips by the highest treatment level.

The most prominent symptom in gills was angiectasis. The Fisher's exact test showed a significantly higher (*p* < 0.05) incidence at all treatments with the exception of 320 µg/L. However, no concentration-effect relationship was obtained. The Armitage trend test indicated no significant (*p* > 0.05) increase in incidence and severity grade of angiectasis up to the highest test concentration of 1000 µg/L. Angiectasis-like alterations can also be induced by the anesthetic phenoxyethanol and during fixation and preparation of the slides, for example, by different fixation delay periods between treatments 22. Because no inflammatory symptoms were detected in parallel, the angiectasis-like alterations were considered to be not related to DCF exposure. The severity grades of this histological alteration were quite different within the four tank replicates of the treatments, for example, severity 2 to 3 were obtained in two tank replicates at 320 µg/L, but in the other two replicates of this treatment none of the fish showed any lesions (see Supplemental Data, Fig. S1). This difference between the replicates within one treatment strongly indicates that DCF was not responsible for the occurrence of this alteration, at least at concentrations up to 320 µg/L. In general, the significance of angiectasis in the absence of inflammatory processes is inconclusive and was deemed to be incidental and not treatment-related in the present study. In conclusion, a toxicologically relevant effect was established in the gills at a nominal concentration of 1000 µg/L DCF, due to the presence and incidence of focal proliferation of interlamellar and chlorine cells, associated with thickened lamellar tips and mononuclear cell foci. The NOEC for histopathological effects was therefore assessed to be 368 µg/L DCF (mean measured, nominal 320 µg/L).

### ELS test with zebrafish

#### Analytics

To confirm the correct dosage of DCF, 33 test solution samples were analyzed on a weekly basis at six dates during the test (for details see Supplemental Data, Table S2). No DCF was detectable in the control treatment. The DCF concentrations in the analyzed test media of nominal concentrations 10 to 1000 µg/L ranged from 91 to 126% of the nominal values. The highest test concentration of 3200 µg/L was not analyzed because this concentration was above the lowest observed effect concentration (LOEC) and thus not relevant for the interpretation of the biological results. As in the trout ELS study, the biological results were related to the nominal concentrations and the NOEC's additionally to the mean measured DCF concentrations.

#### Hatching rate

Hatching was completed on day 4 postfertilization in the control and at all test concentrations up to 1000 µg/L. At 3200 µg/L, a few larvae hatched on day 5. The mean hatching rate in the control was 87 ± 12% (mean ± SD, Fig. 1). For detailed results of all population parameters, see also Supplemental Data, Tables S5 and S6. The mean hatching rate in the test concentrations up to and including 1000 µg/L ranged from 96 and 110% of the control value without statistically significant differences to the control. At 3200 µg/L, the mean hatching rate was statistically significantly reduced to 37% of the control. Therefore, the NOEC for hatching rate was 1131 µg/L (mean measured, nominal 1000 µg/L).

#### Development rate

In all treatments up to and including 1000 µg/L, the development rate of the larvae from egg fertilization to hatching of the larvae was in the same range as that of the control (99 to 101%), without statistically significant differences to the control. At 3200 µg/L, the mean development rate was statistically significantly reduced to 89% of the control. The NOEC for the development rate of the eggs was therefore 1131 µg/L.

#### Survival

The survival rate of the control fish until test end was 87 ± 8% (mean ± SD), demonstrating suitability of the test conditions. Mean survival rates up to 320 µg/L ranged from 78 to 91% and did not differ significantly from the control (Fig. 2). The fish at these concentrations were healthy and showed normal behavior and no visible abnormalities. At 1000 µg/L, the mean survival rate was reduced to 68% (corresponding to approximately 78% of the control value). This decrease was statistically still not significant due to the large variability of the survival rate in the tank replicates, ranging from 39 to 93% in this treatment. At the highest concentration of 3200 µg/L, survival was statistically significantly reduced to an average of 25% compared to the control. Based on the statistical analysis, the NOEC for survival is 1000 µg/L. However, we assessed the mortality of more than 30% at 1000 µg/L to be biologically relevant, and therefore we judged the NOEC for survival of zebrafish to be 336 µg/L (mean measured, nominal 320 µg/L).

#### Growth

Mean body length, body wet, and dry weight were slightly, but statistically not significantly lower compared to the control at the lowest test concentration of 10 µg/L. At the highest test concentration of 3200 µg/L, a clear reduction in mean body length to 68% of control and in mean wet weight to 39% of control was obtained. Determination of the mean dry weight was technically not possible due to the very low number of surviving fish at this concentration. However, at the middle test concentrations of 32, 100, 320, and 1000 µg/L, the mean body wet weight was moderately reduced, but without showing a dose response (by 26 to 34%, on average 29%) compared to the control (Fig. 3 and Supplemental Data, Table S6). The body wet weight reduction was statistically significant at 32, 100, and 1000 µg/L, but not at 320 µg/L. The mean body dry weight at 32 to 1000 µg/L was equally lowered by 25 to 27% (on average 25%) compared to the control. This reduction was statistically significant at all of these concentrations. The body length at 32 µg/L up to 1000 µg/L was only slightly reduced by 9 to 13% (on average 11%) compared to the control. This reduction was statistically significant at 32 and 320 µg/L, but not at 100 and 1000 µg/L. Thus, in the concentration range of 32 to 1000 µg/L, a plateau was obtained where the mean fish size was very similar and independent from the test concentrations. This plateau indicates that for both growth parameters length and weight, no dose-response relationship was obtained within a wide concentration range. No plausible reason is known to us, which could explain this “plateau effect,” that is, DCF affecting the size of zebrafish but not of rainbow trout. Due to the absence of a clear dose-response in the wide test concentration range of 32 to 1000 µg/L, and because clearly no growth inhibition was obtained in the rainbow trout study at these concentrations, we believe that the most probable NOEC for zebrafish growth is between 320 and 1000 µg/L (nominal concentrations). For a detailed rationale of our assessment, refer to the *Discussion* section.

### BCF test with rainbow trout

All BCF values, the steady state BCF_SS_, the kinetic BCF_K_, and the lipid normalized BCF_L_ (for definitions refer to the *Materials and Methods* section) ranged between 2 and 9 in both treatments (Table 2). All BCF's were based on total radioactivity in parent equivalents in fish (µg equivalents/g) and the average concentration of total radioactivity in water. During the accumulation period, total radioactivity levels in the test solutions remained sufficiently constant and amounted on average to 2.09 ± 0.14 µg/L and 18.70 ± 1.05 µg/L (means ± SD) in the low dose tank and high dose tank, respectively (105 and 94% of the nominal concentrations). Chromatographic analysis by HPLC showed that the radioactive parent DCF represented 97 to 99% of the radioactivity measured in the test solutions during the uptake phase. One additional minor peak was observed, accounting for maximally 3.1% (low dose) and 1.4% (high dose) of the radioactivity in the water. Radioactivity levels in the water of the control and in the exposure tanks during the depuration phase were <LOQ (0.048 µg/L) at all sampling dates. All fish were healthy and showed normal behavior and no visible abnormalities.

**Table 2:** Bioconcentration of diclofenac in present fish study

Parameter	Low concentration (2.1 µg/L)	High concentration (18.7 µg/L)
BCF_SS_	5	3
BCF_K_	2	2
BCF_L_	9	5
Depuration half-life DT50 (d)	1.1	0.9

BCF = bioconcentration factor; BCF_SS_ = steady state bioconcentration factor (calculated from fish concentrations at steady state plateau); BCF_K_ = kinetic bioconcentration factor (calculated from fitted uptake and depuration rate constants); BCF_L_ = lipid normalized bioconcentration factor (BCF_SS_ normalized to 5% fish lipid content).

**Fig. 5:**
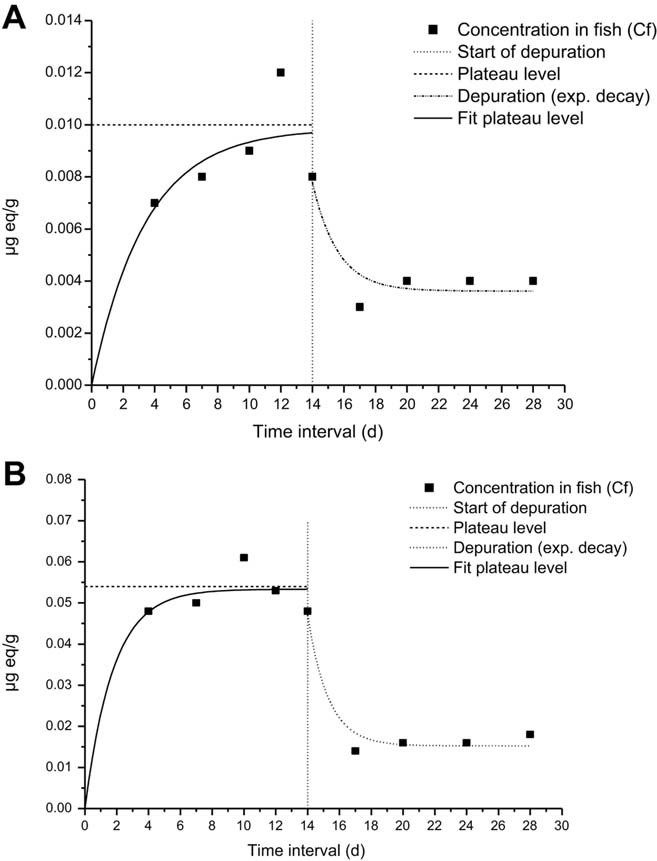
Total radioactive residues in fish (µg equivalents/g fish) of the low dose (**A**; 2.1 µg/L) and high dose (**B**; 18.7 µg/L) treatment with diclofenac during 14 d of exposure and 14 d of depuration.

The radioactive residues in whole fish increased rapidly during the uptake phase. A plateau level was reached within 14 d (Fig. 5). The radioactivity in fish at plateau level was 0.010 µg equivalents/g in the low dose treatment (2.1 µg/L), and 0.054 µg equivalents/g in the high concentration (18.7 µg/L). Due to the negligible accumulation in fish, the concentrations were determined as total radioactivity (parent equivalents), and based on OECD test guideline 305, no specific chromatography was conducted to separate parent DCF and metabolites in the fish. Radioactivity in fish decreased rapidly during depuration, with a depuration half-life (DT50) of 0.9 to 1.1 d (Table 2). At the low dose, extremely low concentrations were measured in the fish at day 17 (day 3 of depuration) until day 28. These values (estimated 0.003–0.004 µg equivalents/g fish) were below the LOQ of 0.005 µg equivalents/g. Also in the high dose treatment, the radioactive residues were depurated to very low concentrations of 0.014 to 0.018 µg equivalents/g at days 17 to 28. These concentrations were only slightly above the high dose LOQ of 0.009 µg equivalents/g fish. In the control tank, all concentrations in fish were <LOQ throughout the study period.

## DISCUSSION

### Bioconcentration in fish

The very low bioconcentration in fish determined in the present study corresponds to the chemical properties of DCF. Diclofenac is an ionisable chemical with a pK_A_ of 3.99 to 4.16 14, 23. Thus, water solubility and octanol-water distribution coefficient are pH-dependent. The water solubility of the DCF salt is distinctly higher than the solubility of pure DCF. The log *K*_OW_ (octanol-water partition coefficient), determined for nonionised DCF at pH 3, is 4.51, which is rather high 14. However, the octanol-water distribution coefficient (log *K*_D_) is only 1.9 at pH 7.0 23 and 1.31 at pH 7.4 14. Thus, for the estimation of fish bioconcentration by the octanol-water distribution coefficient, the *K*_D_ values of DCF at the environmentally relevant pH range of 6 to 9 should be taken into account.

Schwaiger et al. 10 determined the bioconcentration of DCF in different organs of rainbow trout after exposure for 28 d. Their BCFs ranged from 69 in muscle to 2732 in liver at 1.0 µg/L, and decreased continuously with increasing test concentration up to and including 500 µg/L. Other authors have published results on the bioconcentration of DCF in the bile, liver, and blood plasma of rainbow trout (summarized in Table 3). Bioconcentration of chemicals in these fish compartments can be a useful indicator for exposure for environmental monitoring purposes. However, for potential biomagnification and secondary poisoning of a chemical within the aquatic food chain, the body burden in whole fish is normally more relevant because fish-eating birds and mammal predators usually consume whole fish. The bioconcentration of DCF in whole fish was therefore measured in the present study.

**Table 3:** Bioconcentration factors of diclofenac in present fish study compared to published studies

Exposure period	Fish species and exposed life stage	BCF determined in	Test concentration (µg/L)	BCF	Reference
10 d	Rainbow trout 1 year	Bile	1.7	320–950	Kallio et al. 18a
10 d	Rainbow trout 1 year	Bile, blood plasma	1.8/43	476/797, 5.7/4.9	Lahti et al. 19b
14 d	Rainbow trout 6 months	Blood plasma, liver	1.6–81.5	4.0, 2.5	Cuklev et al. 25b
14 d	Rainbow trout young (1.1–1.2 g wet wt)	Whole fish	2.1/18.7	5/3	Present study^c^
21 d	Rainbow trout adult	Bile	0.5/5/25	657/534/509	Mehinto et al. 13b
28 d	Rainbow trout 22 months	Liver	1.06–501.2	12–2,732	Schwaiger et al. 10b
		kidney		5–971	
		gills		3–763	
		muscle		0.3–69	

a BCF related to sum of diclofenac and metabolites.

b BCF related to diclofenac only.

c BCF related to sum of diclofenac and metabolites (total radioactivity measured).

BCF = bioconcentration factor.

The steady-state plateau was reached within a short period of approximately 10 to 14 d, and the small amounts of DCF taken up by the fish were rapidly depurated, with a half-life DT50 of approximately one day. Similar fast depuration of DCF in fish was also observed in other studies 19, 24. The very low concentrations measured in the fish at the end of the depuration period made it difficult to assess if small amounts of radioactivity still remained in the fish after the 14-d depuration phase, as indicated by the fitted depuration curve. One reason for small amounts remaining might be enterohepatic circulation of DCF in fish, as was described by Hoeger et al. 24.

All BCFs in the present study were below 10 and showed no concentration-dependency within the tested concentration range of 2 to 19 µg/L. Also, Lahti et al. 19 and Cucklev et al. 25 found no concentration-dependency for bioconcentration of DCF in fish plasma and liver. According to the technical guidance document (no. 27) for deriving environmental quality standards (https://circabc.europa.eu/d/a/workspace/SpacesStore/0cc3581b-5f65-4b6f-91c6-433a1e947838/TGD-EQS%20CIS-WFD%2027%20EC%202011.pdf), evidence for a relevant bioaccumulation is indicated at BCF's ≥ 100. Thus, the very low BCF values of the present study indicate no potential of DCF to bioconcentrate in fish. Consequently, also, the potential of DCF for secondary poisoning by fish is low.

### Fish toxicity

Many studies of the acute or (sub-)chronic toxicity of DCF to aquatic organisms have been published in the past. Toxicity data for DCF after short-term exposure (up to 4 d) have been reported for several aquatic species including algae, water plants, crustacean, bivalves, and some fish species 5–9. Overall, fish seem to be most sensitive. The acute toxicity of DCF to juvenile fish is relatively low, with 96-h LC10 of 8 mg/L for Japanese medaka 26 and 96-h LC50 of 167 mg/L for zebrafish 27. As the measured DCF concentrations in surface waters are mainly in the range of ng/L to low µg/L, the acute risk for the different organism groups and trophic levels in the aquatic environment can be neglected. Human pharmaceuticals are typically discharged continuously from patient excretion via sewage effluent to surface waters. Thus, chronic exposure is much more relevant for the environmental risk assessment (ERA) than pulsed short-term exposures of, for instance, pesticides. Consequently, chronic toxicity data, as actually required by the European Medicines Agency (EMA), are needed for the aquatic ERA of pharmaceuticals 28.

Most chronic fish toxicity studies with DCF used histopathology, sub-cellular, or other endpoints. The NOEC's cover a broad range of concentrations. Histopathological findings (Table 4) often were the most sensitive endpoints. Kidney, liver, and fish gills are possible target organs for DCF, due to their detoxification ability and/or direct contact to xenobiotics. For DCF, histopathological changes in the kidney are considered likely, given the population decline of Asian vultures after consuming carcasses of DCF-treated cattle, resulting in irreversible short-term damage to their kidneys 29–31. The liver may be exposed to increased local concentrations of DCF during detoxification processes. The gills also are a potential target organ, due to the extensive exposure to xenobiotics and their high permeability for chemicals in the water.

**Table 4:** NOEC's (in µg/L) for histopathological effects of diclofenac in fish organs

	Schwaiger et al. 10	Hoeger et al. 12	Mehinto et al. 13	Praskova et al. a	Present study
Liver	≥500	0.5	≥25	≥60,000	≥1,000
Kidney	1	5	1	≥60,000	≥1,000
Gills	1	5	—	≥60,000	320
Skin	—	—	—	≥60,000	—
Gastro-intestinal tract	≥500	—	1–5	—	—
Spleen	≥500	—	—	—	—
Exposure period	28 d	21 d	21 d	28 d	95 d
Fish species	Rainbow trout	Brown trout	Rainbow trout	Zebrafish	Rainbow trout

a E. Praskova et al., University of Veterinary and Pharmaceutical Sciences Brno, Department of Veterinary Public Health and Toxicology, Brno, Czech Republic, unpublished manuscript.

NOEC = no observed effect concentration; — = not examined; ≥ = no effect up to and including the highest test concentration.

Other sublethal fish studies conducted with DCF resulted in distinctly lower NOEC's compared to the results of the present studies (Table 4). After the exposure of adult rainbow trout for 28 d to DCF (1.0–500 µg/L), Schwaiger et al. 10 reported statistically significant histopathological effects in the kidney and gills starting at 5.0 µg/L (NOEC 1.0 µg/L). No histological effects were obtained in that study in liver, intestine, and spleen (using light microscopy). The same fish were analyzed by Triebskorn et al. 11, 32 at the ultrastructural level using electron microscopy. In that study, subcellular cytopathological effects on liver, kidney, and gills were determined already at 1.0 µg/L. Hoeger et al. 12 exposed 18 month-old brown trout to DCF (0.5, 5.0, 50 µg/L) for 21 d and conducted histopathology in gills, trunk kidney, and liver. Mild to moderate effects were observed in all organs. A NOEC of 0.5 µg/L was set, based on the effects in the liver. However, neither Schwaiger et al. 10, Triebskorn et al. 11, 32, nor Hoeger et al. 12 found a clear dose-response for their histopathological findings. Therefore, a clear-cut NOEC is difficult to identify from these results. Mehinto et al. 13 exposed juvenile rainbow trout to DCF (0.5, 1, 5, 25 µg/L) for 21 d. Histopathological evaluations showed tubular necrosis in the kidney and alterations in the intestine, but no morphological changes in the liver. Based on the histological findings, they proposed a NOEC of 1.0 µg/L. However, Praskova et al. (E. Praskova et al., University of Veterinary and Pharmaceutical Sciences Brno, Department of Veterinary Public Health and Toxicology, Brno, Czech Republic, unpublished manuscript) did not find any histopathological effects in a 28-d toxicity study with juvenile zebrafish and exposure at 0.02 to 60 mg/L. Taken together, the data in the available literature show inconsistent results that make it difficult to determine a no-effect level for DCF.

The low threshold effect levels of 0.5 to 1.0 µg/L obtained in some of these published studies could not be confirmed in the present ELS studies with rainbow trout and zebrafish despite the very long exposure periods (compared to other studies) and the use of typically very sensitive early life stages, and despite the combination of both histopathology and population-relevant endpoints in the trout study. Histopathological alterations were found in trout gills at 1084 µg/L, but no relevant histopathological symptoms were observed in kidney and liver up to the highest test concentration. However, none of the population-relevant (apical) endpoints such as hatching, development, growth, or survival were affected up to and including the highest test concentration of 1084 µg/L. The mean body weight and length of the trout at the higher test concentrations were even slightly larger compared to the control fish. Consequently, the present study with rainbow trout demonstrates an overall NOEC (including all monitored population-relevant endpoints as well as histopathology of the potentially targeted organs gills, kidney, and liver) at 320 µg DCF/L.

While the present ELS test with rainbow trout demonstrated that DCF clearly had no inhibitory effect on fish growth up to and including the highest test concentration of 1000 µg/L, the growth effects of DCF on zebrafish in the present study were less straightforward to interpret. The moderate but more or less constant size reduction of the zebrafish at the end of the study over a wide concentration range (32–1000 µg/L) can be interpreted in two different ways. Based on the statistical results, interpretation A suggests a NOEC for the growth of the zebrafish at 10 µg/L, because the mean length and weight at 32 µg/L were statistically significantly reduced. However, the mean values at the next higher test concentrations were not always significantly different from the control. Interpretation B considers the moderately reduced zebrafish growth from 32 to 1000 µg/L as an artifact of some sort, which is not a treatment-related, repeatable, adverse effect of DCF.

In our opinion there are three reasons to favor interpretation B. The first reason is the absence of a dose-response relationship in the wide concentration range of 32 to 1000 µg/L. No plausible toxicological explanation is known to us, which could explain this plateau effect of a constant inhibition in growth over such a large concentration range. If DCF at a concentration of 32 µg/L does produce an adverse effect on fish growth, this effect would be expected to be progressively larger at 100, 320, and 1000 µg/L DCF, respectively. In contrast, fish size at these concentrations showed a typically normal pattern for fish growth, with no trend to decrease with increasing test concentrations. At the next higher test concentration of 3200 µg/L, both the mean length and weight of the zebrafish were clearly reduced (reduction in length by 32%, in wet wt by 61% compared to the control). Thus, a typical dose-response relationship was observable only between 1000 and 3200 µg/L. The second reason supporting interpretation B comes from the results of a recently submitted 28-d growth study with zebrafish according to the OECD test guideline 215 33. Praskova et al. (E. Praskova et al., University of Veterinary and Pharmaceutical Sciences Brno, Department of Veterinary Public Health and Toxicology, Brno, Czech Republic, unpublished manuscript) found no significant inhibitory effect of DCF on the growth of zebrafish up to 5 mg/L. Once confirmed by peer review, this result would strongly support our conclusion that it is unlikely that DCF inhibits the growth of zebrafish in the present ELS study within the concentration of 32 to 1000 µg/L. The third reason supporting interpretation B comes from the findings of the ELS study in rainbow trout, where clearly no inhibitory effect on growth was obtained up to the highest test concentration of 1000 µg/L. A 100-fold difference in growth sensitivity of two teleost fish species toward DCF resulting in a NOEC of 10 µg/L in zebrafish and a NOEC of ≥1000 µg/L in rainbow trout seems very unlikely. No reason is known to us to explain this apparently extreme susceptibility of zebrafish compared to rainbow trout.

In general, for a chemical like DCF, with a receptor mediated, specific mode-of-action (MOA), similar sensitivities can be expected for all teleost fish. For example, all species of fish investigated to date with the human pharmaceutical ethinylestradiol displayed relatively similar sensitivities 34, as a consequence of all fish species possessing estrogen receptors, which are the key targets for that particular pharmaceutical. In humans, DCF acts essentially as a cyclooxygenase (COX) inhibitor. If DCF also acts via this specific MOA in zebrafish and trout (which is likely, due to the largely conserved structure of COX genes in zebrafish 35, trout 36, and mammals, respectively), then we would expect both species to demonstrate relatively similar sensitivities to DCF. Based on the three reasons discussed above we consider the moderately reduced zebrafish growth in the concentration range of 32 to 1000 µg/L as an artifact of some sort, but not as a treatment-related, repeatable, adverse effect of DCF. In our view, a real adverse effect on growth may have been present first at 3200 µg/L. Our assumption is that a faster growth of the zebrafish in the control group was the reason for the difference in fish size in the concentration range of 10 to 1000 µg/L. A faster growth rate can happen by an unknown mechanism as a consequence of the test design or even by chance. For example, Owen et al. 37 reported very similar findings to those observed in the present zebrafish study. They tested the effect of clofibric acid in a fish growth study with rainbow trout according to OECD test guideline 215 33 and observed a significant reduction in fish weight and growth rate already at the lowest test concentration of 0.1 µg/L. A very similar inhibition of the growth rate by approximately 50% was obtained at all test concentrations up to 10,000 µg/L. Due to this unexpected result without any dose-response, Owen et al. 37 repeated parts of the study by testing the lower test concentrations again but this time with more replicates per treatment, to increase the statistical power. In the study repeat no adverse effects on fish growth were obtained, that is, the effects observed in the first test were not reproducible. After a detailed evaluation of the results of these two tests, Owen et al. 37 came to the conclusion that the results of the first test “could be attributed primarily to an exceptionally fast growth rate in the control fish.”

The reason for the differences in the growth of the zebrafish in the middle test concentration range of the present ELS study remains unknown. However, when the present zebrafish and rainbow trout studies, the published genetic evidence, and the emerging zebrafish study of Praskova et al. (E. Praskova et al., University of Veterinary and Pharmaceutical Sciences Brno, Department of Veterinary Public Health and Toxicology, Brno, Czech Republic, unpublished manuscript) are considered together, we strongly believe that the reduced growth in the zebrafish study in the concentration range of 32 to 1000 µg/L could be attributed primarily to an exceptionally fast growth in the control fish compared to the growth in the treated fish. This leads us to the conclusion that DCF has, with high probability, no inhibitory effect on fish growth up to at least 320 µg/L. This proposed NOEC of 320 µg/L for zebrafish is identical to the overall NOEC of the present trout study. The only difference is the NOEC trigger in zebrafish, which was a reduction in survival and possibly in growth at 1000 µg/L, while the trigger in the trout study were the histopathological findings.

A large discrepancy remains between the NOEC's that have been postulated in some of the published studies and those determined in the recent toxicity tests with rainbow trout and zebrafish. The NOEC of the present ELS test in rainbow trout is up to 640-fold higher than the lowest postulated NOEC of 0.5 µg/L 12. The reasons for this discrepancy cannot be explained with absolute certainty by the present study. Comparing the published histopathological effects of DCF, they show several inconsistencies. The interstudy impact of DCF on different fish organs and the specified symptoms are partly contradictory (Table 4). For example, Mehinto et al. 13 found no pathological effects in trout liver, but did report effects in the kidney and in the intestine. Schwaiger et al. 10 described the most prominent effects in gills, followed by kidney, but no effects in trout liver or gastro-intestinal tract. In contrast, Hoeger et al. 12 found the strongest effects in liver, followed by gills and kidney. All these studies were conducted with brown or rainbow trout. It seems unlikely that the differences obtained were caused by a different mode of action of DCF in these related trout species. The inconsistencies may, however, have been caused by other factors. One factor may be the different classification systems used to evaluate incidence and severity grade of potential symptoms. Overall assessment based on severity can be biased, for example, by combining minimal or slight findings with moderate symptoms in one coarse severity class. In addition, differences between the treatments and the control can only be assessed in a reliable manner if the baseline frequency of symptoms in the control is known 38. The relatively low number of histopathologically analyzed control fish in the earlier published studies leaves some doubts as to whether such baseline frequencies were reliably quantified and considered. High experience in histopathology is needed to avoid misdiagnosis of results because histopathological findings can be influenced by many factors, such as biological variability, diseases, parasites, or other stress in the test fish, for example from too high fish density. Additionally, histopathology leaves room for subjective interpretation. The semi-quantitative results, obtained through histopathology, require special statistical methods, and most importantly, expert judgement to avoid misdiagnosis based on over-interpreted, isolated findings. This point can be exemplified by the difficulties and recent efforts to harmonize the interpretation of fish gonad pathology findings. Fish pathology experts recently developed an OECD guidance document for the technical preparation and histopathological evaluation of fish gonads 39. An official guidance document for histopathology in fish organs other than gonads is, however, still missing. Such technical guidance and a validated rating system might help to avoid the inconsistencies obtained in the case of DCF fish pathology. Presently, it is very difficult to link histopathological results obtained in fish studies to adverse effects on a fish population level. To allow such an extrapolation from histopathological findings to population-relevant apical endpoints (i.e. development, growth, survival, or reproduction), studies are needed which include both high quality histopathology as well as population-relevant endpoints. Such studies are still scarce for DCF.

In Europe, environmental quality standards are proposed as legally binding target levels for selected surface water contaminants. The technical guidance document (no. 27) for deriving environmental quality standards (https://circabc.europa.eu/d/a/workspace/SpacesStore/0cc3581b-5f65-4b6f-91c6-433a1e947838/TGD-EQS%20CIS-WFD%2027%20EC%202011.pdf) states that test results based on endpoints of whose relationship to effects at the population level is uncertain are unsuitable and should not be used for environmental risk assessment-based decision making. According to this guidance document, histopathological data (with the exception of gonad histology), or findings on a sub-cellular level such as changes in enzyme induction or gene expression, belong to this group of endpoints with unclear population relevance. In summary, histopathological symptoms or sub-cellular endpoints should for now only be used as indicators for further evaluation, but should not be used as decision criteria in ERA processes.

So far, there is limited information available on the effects of long-term exposure of DCF to aquatic organisms. No data from an OECD test guideline 210 ELS test with standardized endpoints or an OECD test guideline 305 fish bioconcentration study were available as required for the ERA, for example, for marketing authorization of human pharmaceuticals in Europe 28. The present studies, conducted according GLP and in accordance with the validated and internationally accepted OECD test guidelines, address these gaps now. Their results should be used in future for the derivation of a robust environmental quality standard for DCF in surface waters under the Water Framework Directive in the European Union.

## CONCLUSIONS

Current aquatic risk assessments for DCF are based on several fish studies using not yet validated endpoints like histopathology or biomarkers. Such endpoints are suitable as an indicator for further evaluation, but are not appropriate for environmental risk assessment based decision-making. Appropriate data recording, reliable analytical investigations, and a standardized experimental setup are other prerequisites for studies used for a reliable risk assessment.

In the present study, the whole-body bioconcentration of DCF in juvenile rainbow trout showed a plateau BCF of <10, indicating that DCF does not have any relevant bioconcentration potential in fish.

In the rainbow trout ELS test, the NOEC including histopathology was determined to be at 320 µg/L. The same NOEC was obtained in the zebrafish test for all endpoints with the exception of growth. The result to the effect of DCF on zebrafish growth was less clear, meaning that, this NOEC can be interpreted as 10 µg/L. However, based on biological and mechanistic reasons mentioned above and in accordance with the findings of an emerging growth study with zebrafish we consider the moderately reduced zebrafish growth rate up to 320 µg/L as an artifact, not a treatment-related, repeatable effect of DCF. This leads us to the conclusion that DCF has, with high probability, no adverse effect on both fish species, rainbow trout and zebrafish, up to 320 µg/L. Because measured concentrations of DCF in European rivers are in the range of ng/L to low µg/L, the NOEC of 320 µg/L indicates a sufficient margin of safety for fish populations. We propose the results of these three high quality benchmark studies should be used now for the risk assessment of DCF.

## SUPPLEMENTAL DATA

Details of the analytical and histopathological methods used and tabled results for analytical and biological data obtained (377 KB PDF).
