# Supplementary material for: DICLOFENAC: NEW DATA ON CHRONIC TOXICITY AND BIOCONCENTRATION IN FISH
Source: Environ Toxicol Chem. 2013 Jan 16;32(2):442–52. doi: 10.1002/etc.2085 (PMC3674524; doi:10.1002/etc.2085)
Supplement: Supplementary file 1 [file etc0032-0442-SD1.pdf]

SUPPLEMENTAL DATA

DICLOFENAC: NEW DATA ON CHRONIC TOXICITY AND BIOCONCENTRATION IN FISH

ULRICH MEMMERT,† ARMIN PEITHER,‡ ROLAND BURRI,‡ KLAUS WEBER,‡ THOMAS SCHMIDT,‡ JOHN P. SUMPTER,§ and ANDREAS HARTMANN*‖

†Eurofins Regulatory, Rheinfelden, Switzerland

‡Harlan Laboratories, Itingen, Switzerland

§Institute for the Environment, Brunel University, Uxbridge, United Kingdom

‖Novartis Pharma Stein, Stein, Switzerland

* To whom correspondence may be addressed (andreas-2.hartmann@novartis.com)

Analytical methods in both fish early life stage (ELS) tests

Instrumental HPLC-MS/MS setup

Autosampler: CTC PAL; injection volume: 2 µL; pump: high pressure gradient with 2 Shimadzu LC-10AD pumps and a Shimadzu SCL System Controller; column: Inertsil ODS-3 (GL Sciences); 2.1 mm x 50 mm, 3 µm; eluent A: 95 vol. water + 5 vol. methanol + 0.1% formic acid; eluent B: 5 vol. water + 95 vol. methanol + 0.1% formic acid; flow rate: 400 µL/minute; ionization mode: ESI; heater gas temperature: 450°C; spray voltage: 5200 V; detector: MDS Sciex API 4000; triple stage quadrupole mass spectrometer; scan mode: Multiple Reaction Monitoring (MRM); retention time: approximately 1.4 minutes.

Gradient:	Minutes	% Eluent A	% Eluent B
	0	40	60
	1.0	0	100
	1.5	0	100
	1.6	100	0
	2.0	100	0
	2.1	40	60
	3.0	40	60

HPLC-MS/MS conditions

Analyte	Ion Polarity	Precursor Ion	Product Ion	Dwell Time [ms]	Collision Energy [eV]
Diclofenac (DCF)	positive	*m/z* 296.0	*m/z* 215.0	200	26

Instrumental HPLC-UV setup

Autosampler: VWR-Hitachi L-2200, Merck-Hitachi L-7200; injection volume: 250 µL; pump: VWR-Hitachi L-2130, Merck-Hitachi L-7100; column oven: Jones/7990, Merck-Hitachi L-7300; column: Inertsil ODS-3, 50 mm x 4.6 mm, 5 µm; eluent A: water + 0.1% phosphoric acid; eluent B: methanol; flow rate: 1 mL/minute; temperature: 40°C in a thermostatic oven; detector: VWR-Hitachi L-2400, Merck-Hitachi L-7400; detection wavelength: 210 nm; retention time: 3.0 to 3.6 minutes.

Gradient:

Minutes	% Eluent A	% Eluent B
0	30	70
5	5	95
8	5	95
8.1	30	70
12	30	70

Validation of HPLC-MS/MS method

Specificity: The biological control samples and an analyzed analytical blank (test water) did not affect the chromatogram at the retention time of diclofenac (DCF). The calibration solutions contained a peak specific for DCF, whose area changed accordingly with known concentration.

Linearity: The R^2 fits of the calibration curves used were 0.9995 to 0.9998. This reflects the linearity of the analytical system within the total calibration range of 1.22 to 30.2 µg DCF/L.

Accuracy (recovery) and precision: Concurrent with the sample analysis, a set of recovery samples accurately fortified at relevant concentrations of DCF was prepared and analyzed. The average recoveries were found to be between 104% and 108% of the spiked values with relative standard deviations between 1.5% and 3.4%. The test sample results were not corrected for recovery. The limit of quantification (LOQ) for DCF in the test and control samples was derived from the lowest calibration solution, which fits into the calibration curve. Taking into account a sample preparation factor of 1.4, the LOQ was 1.71 µg DCF/L (zebrafish test) and approximately 2 µg DCF/L (trout test).

Validation of HPLC/UV method

Linearity: The R^2 fits of the calibration curves used were between 0.9990 and 1.000. This reflects the linearity of the analytical system within the total calibration range of 43.4 to 10700 µg/L.

Accuracy (recovery) and precision: Concurrent with the sample analysis, a set of recovery samples accurately fortified at relevant concentrations of DCF was prepared and analyzed. The average recoveries were found to be between 83% and 112% of the spiked values with relative standard deviations between 0.5% and 9.9%. The test sample results were not corrected for recovery.

Calculation of the analytical results

From the calibration curve, the concentration x of the test substance in an injected sample was calculated by Equation 1:

$$x = \frac{y - a}{b} \qquad (1)$$

where: x: concentration of the test substance in injected sample [µg/L or mg/L]

y: peak area of the test substance in injected sample [counts]

a: y-axis intercept

b: slope

The concentration of the test substance in a sample was calculated by Equation 2:

$$c = x \times F \qquad (2)$$

where: c: concentration of the test substance in the sample [µg/L or mg/L]

x: concentration of the test substance in injected sample [µg/L or mg/L]

F: sample preparation factor

The concentration determined in a test sample or application solution as percentage of the nominal concentration was calculated by Equation 3:

$$\%\ \text{nominal} = \frac{c}{c_{nom}} \times 100\% \qquad (3)$$

where: c: determined concentration in the sample [µg/L or mg/L]

c_{nom}: nominal concentration in the sample [µg/L or mg/L]

The recovery rate of a recovery sample was calculated by Equation 4:

$$R = \frac{c}{c_{fort}} \times 100\% \qquad (4)$$

where: R: recovery rate

c: determined concentration of the test substance in the recovery sample [µg/L or mg/L]

c_{fort}: fortified concentration of the test substance in the recovery sample [µg/L or mg/L]

Analytical methods in the fish bioconcentration (BCF) test

Instrumental setup for total radioactivity

Liquid scintillation counters Packard TRI-CARB 2500 TR and 2900 TR equipped with DPM and luminescence options.

All measurements were performed after scintillation background correction and all samples were determined at least in duplicate. The following scintillation fluids and reagents were used: A Irga-Safe Plus (PerkinElmer), B Solvable (PerkinElmer). Aqueous samples: tank water samples (10 mL) were mixed with 10 mL of scintillation fluid A before measurement. Fish samples:

solubilized fish samples of 1.0 mL (containing 100 mg tissue) were measured in 10 mL scintillation fluid A.

Instrumental setup for [^{14}C]DCF and all other radioactive fractions in the test solutions

HPLC; pump: Merck-Hitachi L-6200; autosampler: Merck-Hitachi AS-2000 A; UV-detector: Merck-Hitachi L-4000; radio detector: Packard Radiometric 500TR; column: Luna C18 (2), 250 x 4.6 mm, 5 µm; pre-column: LiChrospher RP18, 4 x 4 mm, 5 µm; temperature: ambient; flow rate: 1.0 mL/min; retention time for diclofenac: 21.8 minutes.

Gradient:

Minutes	% Eluent A	% Eluent B
0	95	5
30	5	95
35	5	95
36	95	5
40	95	5

Lipid measurement in fish

The fish pools were homogenized in a Waring Blendor in the presence of dry-ice, and the homogenized tissue samples were dried with Hydromatrix drying reagent. Thereafter, the homogenates were extracted in two cycles in the ASE (Accelerated Solvent Extraction) system using hexane. The hexane solvent extracts were evaporated under as gentle steam of nitrogen. The lipid pellets were dried at 105°C to a constant weight.

Calculation of BCF's

The steady state BCF_{SS} is the ratio of the concentration in fish (C_f) at the plateau level (i.e. at the last three successive fish sampling dates) to the measured concentration in water (C_w). The kinetic bioconcentration factor (BCF_K) was calculated by fitting the uptake rate constant k_1 and the depuration rate constant k_2 by the non-linear parameter estimation program Origin (MicroCal). All bioconcentration factors were based on the concentration of total radioactivity in parent equivalents in fish (µg equivalents/g fish) and on the average concentration of total radioactivity (C_w) during exposure. The calculations were done according to OECD test guideline 305:

$$BCF_{SS} = \frac{C_f \ \ at\ steady - state}{C_w\ at\ steady - state}$$

$$BCF_K = \frac{k_1}{k_2}$$

where:

C_f = concentration in fish

C_w = concentration in water

k_1 = uptake rate constant

k_2 = depuration rate constant

Histopathological evaluation in the ELS test with rainbow trout

Twenty fish (five per tank replicate) from each test concentration and from the control were randomly selected for histopathological examination of the liver, kidney and gills at the end of the exposure period. These organs were examined for any lesion or alteration.

The symptoms obtained are listed below. No further lesions or alterations were observable in these organs. Furthermore, these lesions included also normal background alterations that were deemed to be normal in control animals.

Liver

- Occurrence of inflammatory cell foci (all cells participating in an inflammatory response)
- Enhanced basophilia (changes in staining abilities in haematoxylin and eosin stained tissues due to increased binding of haematoxylin as an indicator of increased synthesis rate)

Kidney

- Hyaline inclusions (small red to pink staining globules in the renal tubular epithelium)
- Single cell necrosis (cellular within living kidney tissue)

Gills

- Proliferation of interlamellar cells (increase in number of specific cell populations within the gill epithelium)
- Proliferation of chloride cells (increase in number of chloride-secreting cells with large number of mitochondria)
- Inflammation
- Angiectasis (dilatation of a blood or lymphatic vessel)
- Thickened lamellar tips (by cellular and connective tissue proliferation)
- Lamellar fusion as a consequence of previous inflammation

- Mononuclear cell foci (lymphoid cells and macrophages)
- Single cell necrosis of interlamellar cells

Analytical results in the ELS tests

Table S1. Analytically measured concentrations of diclofenac (DCF) in the test solutions of the rainbow trout ELS study

Nominal test concentration	Mean measured concentration of DCF (arithmetic mean)			
(µg/L)	(µg/L)	(% of nominal)	No. of analytical measurements	Dates of analyses (experimental days)
control	<LOQ[a]	-	6	0, 4, 26, 47, 64, 78
3.2	3.1	98	2	0, 4
10	9.7	97	2	0, 4
32	33	102	15	0, 4, 14, 19, 26, 35, 43, 47, 57, 64, 69, 78, 84, 90, 95
100	103	103	15	0, 4, 14, 19, 26, 35, 43, 47, 57, 64, 69, 78, 84, 90, 95
320	368	115	15	0, 4, 14, 19, 26, 35, 43, 47, 57, 64, 69, 78, 84, 90, 95
1000	1084	108	22	0, 4, 8, 14, 19, 22, 26, 28, 33, 35, 43, 47, 50, 54, 57, 61, 64, 69, 78, 84, 90, 95

[a] LOQ (limit of quantification) was approximately 2 µg DCF/L.

Table S2. Analytically measured concentrations of diclofenac (DCF) in the test solutions of the zebrafish ELS study

Nominal test concentration (µg/L)	Mean measured concentration of DCF (arithmetic mean) (µg/L)	(% of nominal)	No. of analytical measurements	Dates of analyses (experimental days)
control	<LOQ[a]	-	3	0, 19, 32
10	11.1	111	6	0, 6, 12, 19, 26, 32
32	36	113	6	0, 6, 12, 19, 26, 32
100	117	117	6	0, 6, 12, 19, 26, 32
320	336	105	6	0, 6, 12, 19, 26, 32
1000	1131	113	6	0, 6, 12, 19, 26, 32
3200	n.a.	-	0	-

[a] LOQ (limit of quantification) was 1.71 µg DCF/L.

n.a. = Not analysed.

Biological results in the ELS tests

Table S3. Survival, hatching and embryo development rate of rainbow trout exposed to DCF

Test concentration		No. of fertilized eggs	Embryo survival (% of fish hatched)				Fish survival from hatch to end of test				Development rate (1/day)			
Nomi-nal	Mean measured													
(µg/L)			mean %	SD	% of contr.	Stat.	mean %	SD	% of contr.	Stat.	mean	SD	% of contr.	Stat.
control		60	98	3	---	---	88	10	---	---	0.0321	0.0007	---	---
3.2	n.a.	60	92	3	94	n.s.	96	4	109	n.s.	0.0312	0.0002	97	s.
10	n.a.	60	100	0	102	n.s.	95	10	108	n.s.	0.0317	0.0001	99	n.s.
32	33	60	98	3	100	n.s.	95	3	108	n.s.	0.0316	0.0001	99	n.s.
100	103	60	95	6	97	n.s.	97	7	110	n.s.	0.0317	0.0002	99	n.s.
320	368	60	98	3	100	n.s.	93	5	106	n.s.	0.0317	0.0006	99	n.s.
1000	1084	60	97	4	99	n.s.	88	7	100	n.s.	0.0315	0.0003	98	n.s.

SD = Standard deviation; Stat. = Statistical evaluation using Fisher`s exact binomial test (survival) and Dunnett t-test (development rate); n.s. = Mean value statistically not significantly smaller than in the control ($p > 0.05$); s. = Statistically significantly smaller than in the control ($p \leq 0.05$); n.a. = Not analysed.

Table S4. Body length and wet weight of rainbow trout measured at the end of exposure to DCF

Test concentration		Body length (mm) [a]				Body wet weight (g) [a]			
Nominal	Mean measured								
(µg/L)		mean	SD	% of contr.	Stat.	mean	SD	% of contr.	Stat.
control		43.9	2.5	---	---	0.77	0.11	---	---
3.2	n.a.	42.1	0.3	96	n.s.	0.70	0.02	92	n.s.
10	n.a.	44.1	1.5	101	n.s.	0.79	0.06	103	n.s.
32	33	44.3	1.7	101	n.s.	0.79	0.05	103	n.s.
100	103	46.1	1.7	105	n.s.	0.85	0.09	111	n.s.
320	368	46.0	1.4	105	n.s.	0.88	0.08	115	n.s.
1000	1084	46.0	1.6	105	n.s.	0.86	0.06	112	n.s.

[a] Medians of body length and body wet weight were calculated per replicate of each treatment in order to reduce the impact of individual outliers. The arithmetic mean and standard deviation shown in this table was then calculated from the median values of the four replicates per treatment.

SD = Standard deviation; Stat. = Statistical evaluation using Dunnett t-test for both endpoints; n.s. = Mean value statistically not significantly smaller than in the control ($p > 0.05$); n.a. = Not analysed.

Table S5. Survival, hatching and embryo development rate of zebrafish exposed to DCF

Test concentration: Nomi-nal (µg/L)	Test concentration: Mean measured (µg/L)	No. of ferti-lized eggs	Embryo survival (% of fish hatched until day 4)				Fish survival from hatch to end of test				Development rate (1/day)			
			mean %	SD	% of contr.	Stat.	mean %	SD	% of contr.	Stat.	mean	SD	% of contr.	Stat.
control		60	87	12	---	---	87	8	---	---	0.288	0.004	---	---
10	11.1	60	83	4	95	n.s.	78	17	90	n.s.	0.288	0.004	100	n.s.
32	36	60	88	11	101	n.s.	91	10	105	n.s.	0.286	0.000	99	n.s.
100	117	60	85	6	98	n.s.	84	7	97	n.s.	0.289	0.004	100	n.s.
320	336	60	95	3	109	n.s.	82	9	94	n.s.	0.288	0.004	100	n.s.
1000	1131	60	90	4	103	n.s.	68	25	78	n.s.	0.292	0.008	101	n.s.
3200	n.a.	60	32	18	37	s.	22	14	25	s.	0.255	0.011	89	s.

SD = Standard deviation; Stat. = Statistical evaluation using Fisher`s exact binomial test (survival) and Welch test (development rate); n.s. = Mean value statistically not significantly smaller than in the control ($p > 0.05$); s. = Mean value statistically significantly smaller than in the control ($p \leq 0.05$); n.a. = Not analysed.

Table S6. Body length and wet and dry weight of zebrafish measured at the end of exposure to DCF

Test concentration		Body length (mm) [a]				Body wet weight (mg) [a]				Body dry weight (mg)			
Nom-inal	Mean measured												
(µg/L)		mean	SD	% of contr.	Stat.	mean	SD	% of contr.	Stat.	mean	SD	% of contr.	Stat.
control		14.3	0.5	---	---	24.2	4.9	---	---	5.1	0.7	---	---
10	11.1	13.8	1.3	97	n.s.	21.3	4.7	88	n.s.	4.9	0.7	96	n.s.
32	36	12.4	1.1	87	s.	15.9	4.0	66	s.	3.8	1.1	75	s.
100	117	13.0	0.4	91	n.s.	17.3	1.3	71	s.	3.8	0.4	75	s.
320	336	12.6	0.5	89	s.	18.0	1.7	74	n.s.	3.8	0.3	75	s.
1000	1131	12.9	0.9	90	n.s.	17.4	4.3	72	s.	3.7	0.5	73	s.
3200	n.a.	9.8	1.0	68	excl.	9.4	1.8	39	excl.	n.a.	n.a.	n.a.	n.a.

[a] Medians of body length and body wet weight were calculated per replicate of each treatment in order to reduce the impact of individual outliers. The arithmetic mean and standard deviation shown in this table was then calculated from the median values of the four replicates per treatment.

SD = Standard deviation; Stat. = Statistical evaluation using Dunnett t-test for all endpoints; n.s. = Mean value statistically not significantly smaller than in the control ($p > 0.05$); s. = Mean value statistically significantly smaller than in the control ($p \leq 0.05$); n.a. = Not analysed; excl. = Treatment excluded from statistical analysis due to low number of 1 - 3 fish per tank replicate (fish growth in tank replicates with significant mortality should not be included in the data evaluation according to OECD test guideline 215).

Histopathological results evaluated in the ELS test with rainbow trout

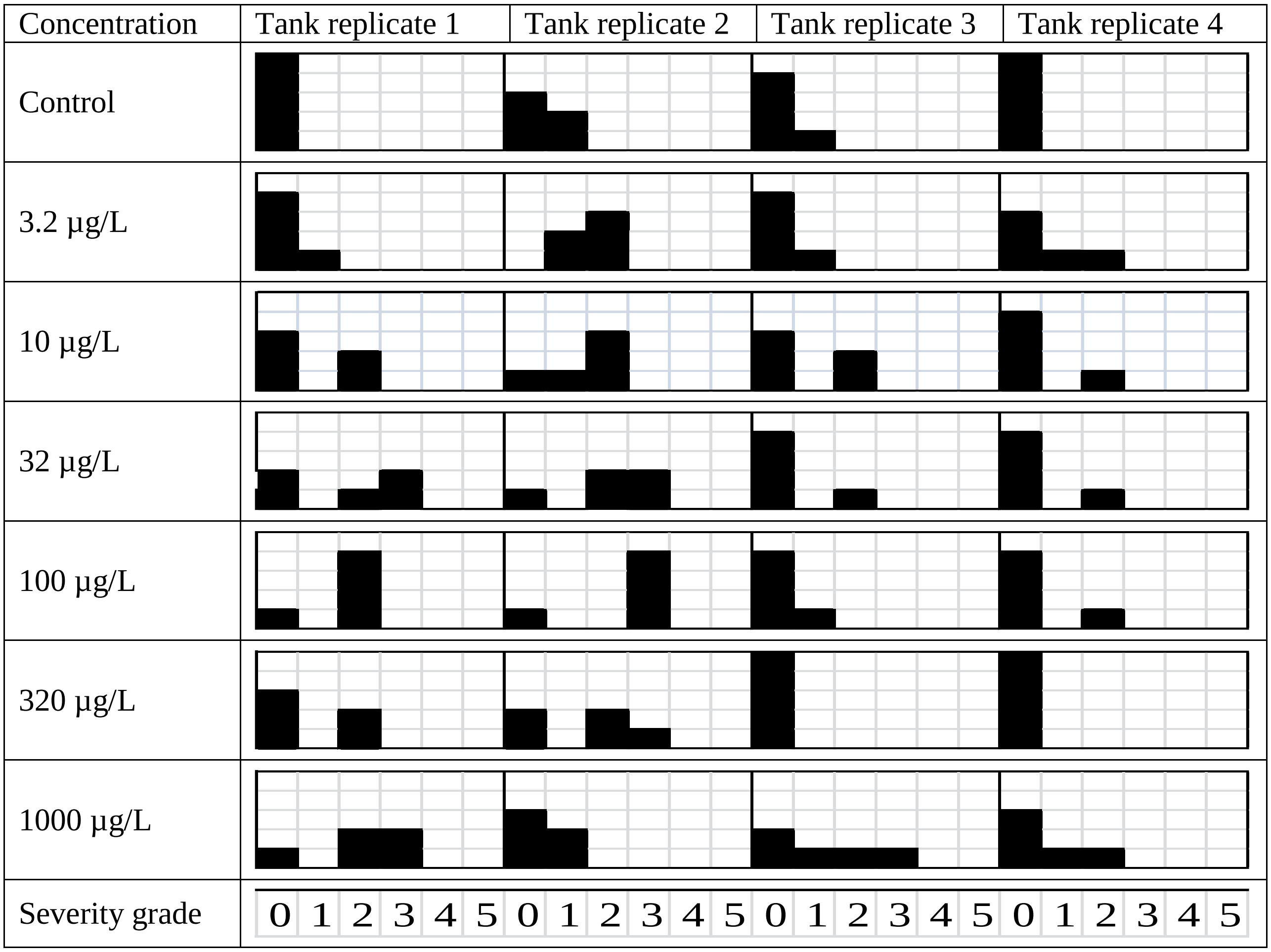

Figure S1. Incidence and severity of angiectasis in gills of rainbow trout treated with diclofenac. Results obtained in the five examined fish per tank replicate are plotted. Incidence is indicated by vertical bar height, severity grade (0 to 5) is displayed horizontally for each replicate. Severity grade 0 = no incidence; grade 1 = <10% (minimal); grade 2 = 10 to 39% (slight); grade 3 = 40 to 59% (moderate); grade 4 = 60 to 79% (marked); grade 5 = 80 to 100% (severe).
